# The critical dimension of memory engrams and an optimal number of senses

**DOI:** 10.1038/s41598-025-11244-y

**Published:** 2025-08-15

**Authors:** Wendy Otieno, Ivan Y. Tyukin, Nikolay Brilliantov

**Affiliations:** 1 Department of Physics, Loughborough University, Loughborough, LE11 3TU UK; 2 King’s College London, London, WC2R 2LS UK; 3 Skolkovo Institute of Science and Technology, Bol’shoi Bulvar 30, Moscow, 121205 Russia; 4 University of Leicester, University Road, Leicester, LE1 7RH UK

**Keywords:** Biological physics, Information theory and computation, Statistical physics, thermodynamics and nonlinear dynamics

## Abstract

In this work, we analyse the fundamental question: how many senses are optimal for memory and learning. To answer this question, we introduce and analyse a novel kinetic model of memory engrams. The model, built on basic general principles and phenomenology, captures the engrams’ emergence and evolution driven by their interaction with external environment, learning, and forgetting. We derive the corresponding kinetic equation governing the dynamics and evolution of engrams over time. We then solve this equation analytically and numerically through Monte Carlo simulations. We observe the formation of a steady state with a steady number of different engrams covering a fraction of the conceptual space. We analyze the impact of the dimension of the conceptual space on the steady state and discover the existence of a critical dimension, at which the number of different engrams is maximal; we provide a theoretical explanation of this observation. If each feature is associated with a different sense, the critical dimension corresponds to an optimal number of senses for a system aiming at keeping the maximal number of different concepts in its memory. We also reveal an apparent tension between the system’s receptivity to new stimuli and concept sharpness—the higher the receptivity, the less sharp the learned concept becomes.

## Introduction

Learning and memory are arguably amongst the most enigmatic, inspiring, and widespread phenomena observed in living organisms. They co-exist and operate across a spectrum of scales including at the molecular level^1^, at the level of single neurons and their networks^2^, and extending to more abstract psychological models and theories^3^. Since 1904, when Richard Semon introduced the term “engram” to describe the neural *substrate* for storing memories^4^, much is known about potential physiological mechanisms supporting memory and memory engrams^5^—sparse ensembles of neurons across multiple brain regions—are viewed as viable hosts of memory^6^. This includes complementary but in no way contradictory evidence that individual neural cells, potentially within an engram, could already be associated with specific memory and concepts^7–10^. In the model presented below, this corresponds to “nested” engrams.

Although the engram concept has been introduced more than a century ago in^4^, the dynamics and evolution of engrams (and hence memories) after their emergence are still not fully understood. A very recent computational and experimental studies^11^ shed some light on how the neural composition and selectivity of single engrams change over a relatively short time. The authors identified specific mechanisms that may be responsible for the emergence of memory selectivity. Notwithstanding this and other works over more than 100 years, many fundamental questions about longer-time behavior of the building blocks of memory—“memory engrams”, remain open. These are:

(i) Is the dimension of the concept space important, and if so then in what respect?
(ii) Can we predict and model what happens with multiple memory engrams evolving and responding to stimulation over longer timescales?
(iii) Do they retain encoding capabilities indefinitely, vanish, dissolve, or aggregate into larger entities encoding super-concepts?
(iv) Does a self-supporting, steady state distribution of engram ensemble exist, and what would be the properties of such a distribution?

In this paper, we present a pathway to answer these open foundational questions. We approach the problem by defining an appropriate mathematical and modelling framework enabling investigation of the long-term behavior of engrams. At the core of the approach is the appropriate reduction of the complexity of detailed biological processes describing the emergence and evolution of memory engrams and concepts to much simpler objects that are mathematically and computationally tractable at large spatial and temporal scales. This model must be based on a very general set of rules and reflect the most prominent features of the engrams, that is, how the engrams interact with the external world through stimuli. In particular, they must reflect the dynamic nature of engrams’ sensitivity to stimuli^11^. According to Ref.^12^ engrams are (i) activated during experience, (ii) undergo structural and functional modifications as a result and (iii) reactivated upon recall of the experience; all these features must be reflected in the model.

Early experimental studies in the field of engram research have focused on engram localization using various techniques such as lesion studies^13–15^, optogenetics^12,16–18^, transgenics^12,19^, pharmacogenetics^12^, immediate early gene (IEG)^20,21^ etc.. These types of studies have paved the way for constructing computational methods such as attractor networks^22,23^, Hopfield networks^24^ and spiking Hebbian networks^25,26^ to simulate memory encoding, consolidation and retrieval. The current state of engram research is the further development of these models as attractor networks focuses on simulating memory stored as stable patterns^27^, spiking Hebbian networks centers on studying the relation between associative memory and sequence generation^26^ and Hopfield networks draws attention to associative memory and pattern recognition where memory is retrieved from information that is partial or corrupted^24^. Despite theoretical and experimental progress in these areas, a sophisticated model which captures the temporal evolution of engrams over time is needed. We formulate kinetic models of memory engrams which describe the size distribution of engrams in *d*-dimensional space. Our approach in solving the equations is employing Monte-Carlo simulations. Not only does our model incorporate the activation/reactivation of engrams through stimuli and reflect changes in engram formation over time, the observed result where higher receptivity of engrams results in learned concepts becoming less sharp aligns with the effects of high receptivity on attractor and spiking Hebbian networks. That is, high receptivity on attractor and spiking Hebbian networks respectively causes convergence to a less distinct attractor state which reduces sharpness and more diffuse synaptic changes which reduces the memory representation’s sharpness. With appropriate setting, our model can simulate manipulation of engrams implemented in loss of function studies (engram impairment leading to the impairment of memory retrieval)^28^, gain of function studies (artificial activation of engrams for memory retrieval)^28^, mimicry studies (artificial introduction of engrams of unreal experience)^28,29^ and studies whereby memory maintenance is showcased by replacing some engrams while others remain stable.

In our model, we assume that a stimulus is defined by a finite set of features, and each feature is encoded by a real number. A stimulus therefore is an element of a linear vector space. We assume that stimuli are comparable with each other in the sense that there is a metric in the space of stimuli enabling us to tell how far one stimulus is away from the other. This property is needed to model how the stimuli are produced in the external environment.

A concept or an engram is an object that can “sense” a stimulus, but its sensitivity is dynamic. Not all stimuli can be sensed or recalled by a single engram. Moreover, the set of all potential stimuli, a single engram can sense or respond to, may change over time. To represent these properties in the simplest way we model engrams as sets in the *d*-dimensional space (associated with a number of stimuli of different nature), whose composition changes over time. The sets representing engrams can grow or shrink over time capturing the engrams’ dynamic sensitivity. The smaller the set is the higher the engram’s sensitivity.

The space in which all engrams are defined is referred as an ambient space and the space in which they dynamically evolve will be called a *conceptual space* or the space of *encoded concepts* stored in memory. The concepts encoded through engrams are not “rigid”—they can partly or completely overlap in the conceptual space, or form nested engrams, when one engram encompasses another one.

To capture stimuli interaction with engrams we follow the recent findings reported in^11^. When a stimulus is sensed by an engram, the engram responds by increasing its sensitivity to that stimulus. In our model, this is equivalent to the engram retaining those elements from its own composition that are sufficiently close to the stimulus and releasing elements that are deemed too far. An activating stimulus may be considered as a “hit” at some point in the conceptual space. That is, it corresponds to an “impression” associated with the set of particular features $(a,b,c,\ldots )$ of an object. If a stimulus “hits” some engram, then an interaction between the stimulus and the engram takes place reflecting the known wisdom—“I see what I know”. If the stimulus is not in any of the existing engrams in the conceptual space, then two alternatives are possible. In the first alternative, the stimulus gets lost without any impact on the conceptual space. In the second alternative, a new engram emerges with the stimulus being among its elements.

These two alternatives give rise to two cases of long-term learning we analyse in our work: “confirming learning” and “learning from scratch”. In the former case, we analyse an already established system, in which a set of engrams/concepts is readily formed and stimuli not hitting a concept are permanently lost. In this regime, we explore the evolution of the system of concepts in response to stimuli. Namely, we study the formation and properties of a steady state. In the latter case, of “learning from scratch”, we analyse the formation of the system of concepts—we assume that a new concept emerges when a few stimuli hit some region in the conceptual space (CS) during a short period of time.

In what follows we present a kinetic model capturing the above description of the evolution and dynamics of engrams and provide both analytical and numerical analysis of its relevant dynamics properties, including the existence and description of steady-states. We also explore how properties of the steady-state depend on the dimension of the conceptual space and whether some dimensions with extreme properties of the conceptual space exist.

The rest of the paper is organized as follows. In Section 2 we present the main results of our work. We start with a one-dimensional model and its analysis, including an analytical solution of the engrams’ steady state and its properties. We then continue with *d*-dimensional models and reveal the existence of a critical dimension of the engrams’ conceptual space at which the number of distinct engrams is maximized. Section 3 describes the methods we used in our exploration, and Section 4 contains a short discussion and a summary of our findings. Additional technical details, including figures (Fig. S1–S5) and analytical derivation of the maximal number of engrams with distinct centres in different settings are provided in Supplementary Materials.

## Results

### Kinetics of engrams in one-dimensional conceptual space

#### Confirming learning

To illustrate our approach, we start with a toy-model—a one-dimensional model of conceptual space. Since there are no prior reasons for the existence of special ’boundary domains’ in the conceptual space, we assume periodic boundary conditions. In particular, we suppose that the conceptual space is reminiscent of a circle or, more generally, a sphere in higher dimensions. This choice is particularly natural for higher dimensions due to the well-know shell concentration^30^ and stochastic separation^31,32^ effects.

In the simplest setting, engrams are one-dimensional segments of different lengths located on a circle in two-dimensional ambient space. Geometrically, the system comprises *n* segments of length $l \in (0,l_{max}]$. Each segment $l_{i}$ $(i = 1,2,...,n)$ is randomly distributed on a circle whose length is *L* and, depending on the value of *L* and/or the total number of segments *n*, may overlap with other segments $l_{j}$ ($i \ne j$).

Stimuli, which will be referred to as the Catching Shots (CS), continuously ’hit’ the concept space at random, at the rate $1/\tau$. They may strike points on the concept space where segments are present or they hit elsewhere. Each catching shot hits the concept space at the average density $1/l_*$, with $l_*=L$, so that, at the time interval $\Delta t$, any point in the interval $\Delta x$ would be hit with the probability $\Delta x \Delta t / l_{*} \tau$. The shot instantly “evaporates” when it hits a point where no segments exist, that is, such hits make no impact on the interaction between future CS and engrams.

The moment a catching shot strikes a segment, the center of the hit segment moves to the point of the catching shot. Next, the length of the displaced segment reduces to size *l* with the probability

1 $$\begin{aligned} P_\textrm{foc}(l) dl= l_{0}^{-1} e^{-l/l_{0}}dl \end{aligned}$$

where $\int _{0}^{\infty } P_\textrm{foc}(l) dl = 1$. This reflects the engram property of sharpening in response to the CS and its affinity to the very last stimulus. That is, the conceptual segment (engram) becomes more focused. Note that Eq. (1) defines the *probability * for a segment length to be equal to *l* after a strike. In general, *l* may exceed the current length of the hit segment $l^{\prime }$. In this case ($l>l^{\prime }$) a more adequate model would read $P_\textrm{foc}(l|l^{\prime })=e^{-l/l_0}\Theta (l^{\prime }-l)(l_0-l_0e^{-l^{\prime }/l_0})^{-1}$, preventing a length increase after a strike. However, if $l_0$ is small, that is, $l_0^2 \ll \alpha \tau l_*$, the probability that $l>l^{\prime }$ would be insignificant and such cases may be neglected (see the definition of $\alpha$ below and the Supplementary Material).

In what follows we will assume that $l_0$ is small and exploit more simple and tractable model, Eq. (1), which is the most simple probabilistic model with an explicit characteristic length. The choice of the exponential density in (1) can additionally be motivated by the maximal entropy principle: it corresponds to the density with the highest entropy, or uncertainty, whose first moment equals $l_0$. This enables modeling ”imprecise” systems in which the system’s expected focusing capability remains constant but the segment size distribution after hits exhibits the highest degree of uncertainty. Further simplifications lead to the case where $P_\textrm{foc}(l) dl = \delta (l-l_0)dl$, with $\delta$ being the Dirac delta function. In the latter setting, the segments always contract to the same length $l_0$ which is hardly realistic.

After shrinkage, the length of the hit segment increases with the rate $\alpha e^{-l/l_{max}}$ where $\alpha >0$ is a parameter controlling the growth rate of the segment and $l_{max}$ is the maximum length a segment can reach (the growth stops when the length reaches $l_{max}$). This reflects engrams’ tendency or predisposition to lose sharpness over time in absence of external stimuli. Segments that have not been hit retain their centres but their size grows with the same length-dependent rate as follows:

2 $$\begin{aligned} \frac{dl}{dt}&= \alpha e^{-l/l_{max}} \Theta (l_{max}-l), \end{aligned}$$

where $\Theta (x)$ is the standard unit Heaviside step-function. Empirical evidence^33^ suggests that forgetting processes could be effectively modelled by sums of exponents with negative rates. A typical pattern is a sharp initial decay followed by a much slower relaxation to the baseline. In our model, we accommodate these phenomenological observations by slowing the growth rate of the segment (the forgetting rate) as the size of the engram grows: the more fuzzy an idea (concept) becomes the slower it loses its sharpness. Instead of employing higher-order linear dynamics to implement multiple exponents, we opted for a single nonlinear ordinary differential equation (2) capable of mimicking a variable rate of decay. Its solutions converge to $l_{max}$ in finite time, indicating that in our model the maximally plausible loss of concept sharpness in the absence of stimuli occurs in finite time too. In principle, one could also consider a more simplified version of the model, with linear forgetting time: $dl/dt=\alpha \,\Theta (l_{max}-l)$. Although offering a computational advantage and simplicity, the simplified model cannot reproduce the slow-fast forgetting dynamics observed in the literature.

**Fig. 1:**
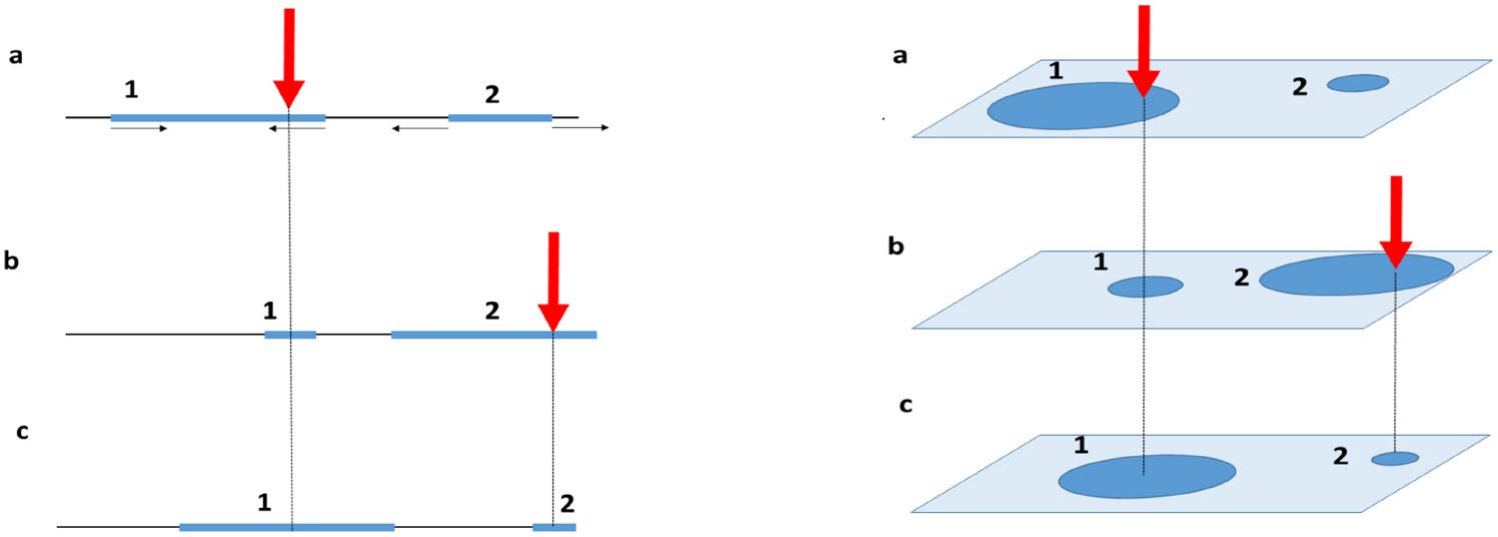
Schematic representation of the model. The blue intervals (or discs) schematically represent engrams. The large red arrows indicate the external stimuli; small black arrows show the direction of engram growth or shrinkage. When a stimulus strikes an engram, (**a**) and (**b**), the engram’s centers shift to the point of the strike (**b**) and (**c**). Without a strike an engram expands. Left panel illustrates 1d system, right panel—2d system.

The general combined model of the system reflects the fact that once a physiological support, or a material carrier, of a concept (and an associated engram) is formed it can evolve and transform but, being the neural substrate for storing memories, can hardly completely vanish in the absence of adverse physiological changes (traumas, illness, etc.)^28^. This is consistent with experimental in-vivo findings revealing that memory formation in developed mice primarily recruits preexisting neural assemblies, rather than forms new neural assemblies^34^. Figure 1 schematically illustrates the model.

Let the number density of segments of the length $l \in [l,l+dl]$, whose centers are located at point *x* at time instant *t*, be characterized by the distribution function *f*(*x*, *l*, *t*), which is normalized to the total number of intervals *n* as

3 $$\begin{aligned} \int _0^{L}dx\int _0^{l_{max}} f(x,l,t)dl =n. \end{aligned}$$

Assuming that distribution of the interval is space uniform, that is, the function does not depend on *x*, we can write for the increment of this function during the time interval $[t,t + \Delta t]$,

4 $$\begin{aligned} {[}f(x,l,t + \Delta t) - f(x,l,t)]dl&= - \frac{l \Delta t}{\tau l_{*}} f(x,l,t) dl - \Delta t dl \frac{\partial }{\partial l} \bigg (\frac{\partial l}{\partial t}\bigg ) f(x,l,t) \nonumber \\&\hspace{1cm} + P_\textrm{foc}(l)dl \int _{0}^{l_{max}} f(x,l',t) \frac{l' \Delta t}{l_{*}\tau } dl'. \end{aligned}$$

The first and second term on the rhs of Eq. (4) respectively give the number of segments of length *l* which disappear during the time interval $\Delta t$ due to the shots and the growth of the intervals. The third term gives the appearance of the segment of length *l* due to the intervals of length $l' \in [0,l_{max}]$ being hit into. Segments of size *l* emerge (from the hit intervals of the length $l'$) with the rate $P_\textrm{foc}(l)dl=e^{-\frac{l}{l_{0}}}dl/l_0$.

For small $\Delta t$, the expansion of the lhs of Eq. (4) yields the equation, which may be called *“engram kinetic equation”*:

5 $$\begin{aligned} \frac{\partial f(l,t)}{\partial t}&= - \frac{l}{\tau l_{*}} f(l,t) - \frac{\partial }{\partial l} \bigg (\frac{dl}{d t}\bigg ) f(l,t) + P_\textrm{foc}(l) \int _{0}^{l_{max}} f(l',t) \frac{l' }{l_{*}\tau } dl'. \end{aligned}$$

Here we take into account the assumption that the density is uniform and does not depend on *x*. The *n*-dimensional generalization of the above equation is presented below. Note that during evolution two or more intervals may intersect and, consequently, may be hit by the same CS landing in the domain in their intersection. In this case, after the hit, the intervals would share the same centre. However, their sizes will be different as being randomly chosen from the appropriate Poisson distribution. Hence they do not merge because they have different size. An alterative case is discussed in Section 2.2.4 (Kinetics of engrams with inhibition).

Using Eqs. (1) and (2) we recast Eq. (5) into the form,

6 $$\begin{aligned} \frac{\partial f(l,t)}{\partial t}&= - \frac{l}{\tau l_{*}} f(l,t) - \alpha \frac{\partial }{\partial l} \bigg [e^{-l/l_{max}} \Theta (l_{max}-l) f(l,t)\bigg ] + \frac{e^{-\frac{l}{l_{0}}}}{ \tau l_{0}} F(t) \end{aligned}$$

where

7 $$\begin{aligned} F(t) = \int _{0}^{l_{max}} f(l',t) \frac{l' }{l_{*}} dl'. \end{aligned}$$

We notice that the average interval length reads,

$$\langle l \rangle =\frac{\int _{0}^{l_{max}} f(x,l',t) l'dl'}{\int _{0}^{l_{max}} f(x,l',t) dl'} =\frac{F(t) l^*}{n/L},$$

where we use the normalization condition (3) for *f*(*x*, *l*, *t*); this yields for $l^*=L$,

$$F(t)= \rho \frac{\langle l \rangle }{L},$$

with $\rho =n/L$ being the density of the intervals.

The stationary solution *f*(*l*, *t*) of the integro-differential equation $(5)$ is independent of *t* along with $F(t)=F$ (a time independent constant). For a space-uniform system *f* does not depend on *x*. Introducing new variable $z = l/l_{max}$ we write the stationary form of $(5)$ for the function $\tilde{f}(z) \equiv f(z\cdot l_{max})$ as

8 $$\begin{aligned} \frac{\partial }{\partial z} \big (e^{-z} \tilde{f}(z) \big ) = -B_{1}z\tilde{f}(z) + B_{2}Fe^{-az} \end{aligned}$$

or

$$\begin{aligned} \frac{\partial \tilde{f}}{\partial z} = -B_{1}e^{z} z \tilde{f}(z) + B_{2}Fe^{(1-a)z} + \tilde{f}(z) \end{aligned}$$

with

$$\begin{aligned} B_{1} = \frac{l_{max}^{2}}{l_{*} \alpha \tau }, \hspace{0.5cm} B_{2} = \frac{l_{max}}{l_{0} \alpha \tau }, \hspace{0.5cm} \text {and} \hspace{0.5cm} a = \frac{l_{max}}{l_{0}}. \end{aligned}$$

**Fig. 2:**
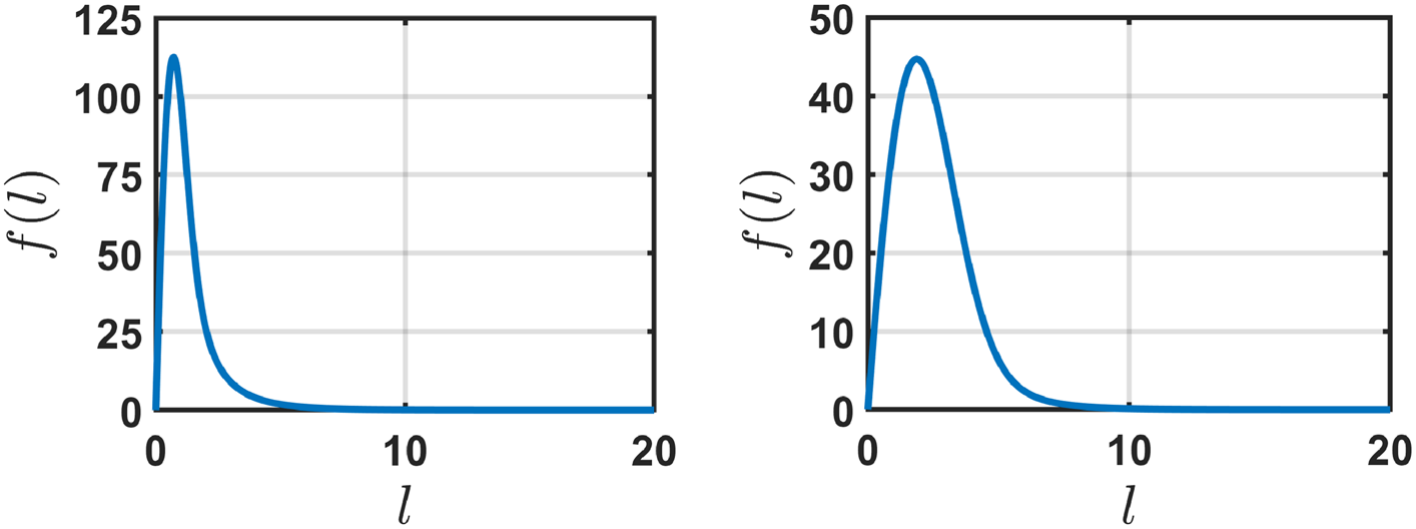
The steady-state distribution for *f*(*l*) for one-dimensional engrams as a function of the engram size *l* for the parameters $(l_{max},\alpha ,\tau ,l_{0},l_{*}) = (10,0.1,1,2,4)$ (left) and $(l_{max},\alpha ,\tau ,l_{0},l_{*}) = (10,1,1,2,4)$ (right) with $f(0) = 0$. *f*(*l*) is a non-monotonic function of *l* which approaches zero for large *l*. The numerical solution of Eq. (6), (7) is indistinguishable from $f(l/l_{max})$ given by Eq. (9), with self-consistently found *F*.

The solution to the steady state equation $(8)$ yields

9 $$\begin{aligned} \tilde{f}(z) = B_{2}Fe^{-B_{1}(z-1)e^{z} + z} \int _{1}^{e^{z}} w^{-(1+a)} e^{B_{1}(\log w - 1)w} dw \end{aligned}$$

for condition $f(0) = 0$. Figure 2 shows the numerical solution of (6), (7) at $t = 1000$ (when a steady-state is achieved) with the initial condition $f(0,l) = 1$ for the parameters $(l_{max},\alpha ,\tau ,l_{0},l_{*}) = (10,0.1,1,2,4)$ (left) and $(l_{max},\alpha ,\tau ,l_{0},l_{*}) = (10,1,1,2,4)$ (right).

As can be seen in the figure, the distribution of the conceptual segments is peaked around the characteristic length corresponding to the size of the segment, $l_0$, immediately after an “impression strike”; the distribution sensitively depends on the learning (hitting) rate $1/\tau$ and the forgetting rate $\alpha$. This fact may be interpreted as follows: the stationary distribution is determined by the trade-off between the focusing of the concepts (which helps to be more specific in classifying the object of the external world) and the ability to better react to the external stimuli (which is larger when “the conceptual segments” have larger size). We expect that during evolution nature “chooses” appropriate values of parameters $\alpha$ and $\tau$ balancing the focusing and forgetting, depending on the learning (hitting) rate. Noteworthy, the oversimplified model with $P_\textrm{foc}(l)=\delta (l-l_0)$ and $dl/dt= \alpha \, \Theta (l_{max}-l)$ also yields a peaked at $l=l_0$ distribution, $f(l)= (F/\alpha \tau l_0) \exp [-(l^2-l_0^2)/(2\alpha \tau l_*)] \Theta (l-l_0)$, with the singularity at $l=l_0$, which makes this model mathematically less convenient, see the Supplementary Material (SM).

#### Learning from scratch

Here we use the following model for the learning from scratch: A new concept, i.e. engram, appears^28^, if *q* successive stimuli hit during a time interval $\tau$ an interval of length $l_s$ of the conceptual space, which is not occupied by other engrams. In this case a new engram—a segment of size $l_s$, centered at the point of the last hit, emerges.

We consider a system with $N = 1000$ segments randomly distributed onto a line, $L = 250$, with a minimum and maximum length of 0.1 and 20 respectively. $\alpha$ and $l_{0}$ are varied independently to investigate the number of distinct centers (NDC) over time after a new segment appears when the same empty region is successively hit $q=3$ times. We observe the behaviour of NDC reaching a steady state for each forgetting rate $\alpha$ and characteristic length $l_{0}$.

**Fig. 3:**
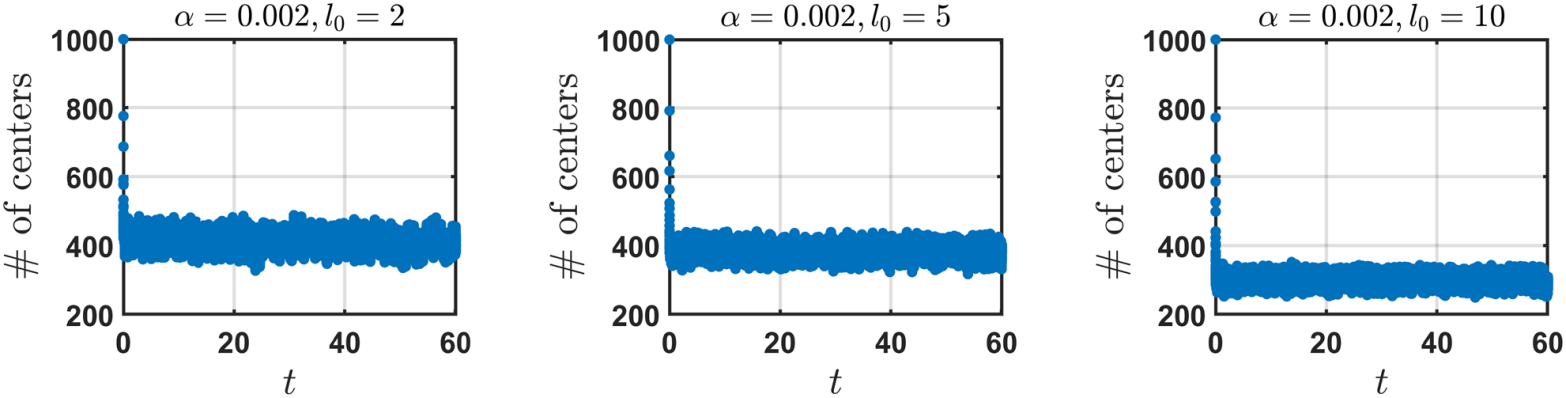
The number of distinct centers (NDC) over time for the model “learning from scratch” for fixed forgetting rate $\alpha = 0.002$ with varied characteristic length $l_{0}$.

NDC initially decreases with time as overlapping hit segments group together to share midpoints. After a significant drop in NDC, it quickly reaches a steady state (see Fig. 3). The probability of introducing a new segment from scratch is much higher for parameters $\alpha$ and $l_{0}$ where NDC is very small. This high probability results to the addition of new segments which will eventually overlap with other segments and group together when hit. The addition of new segments can also lead to center sharing segments breaking apart and forming new centers with the added segments. $\alpha$ controls the growth of segments after the size of the hit segments are changed. For sufficiently small $l_0$, the number of segments introduced into the system grows as $\alpha$ increases. For small $\alpha$ and varied $l_{0}$, NDC decreases with increasing $l_0$. At large $l_0$, NDC is at its minimal value. In addition to that, the number of empty regions reduces, as $l_0$ is large, which in turn reduces the number of segments introduced to the system via successive hits. For larger $\alpha$ and varied $l_{0}$, the value of NDC is small and similar for each $l_0$ however the number of segments that are introduced decreases.

These results clearly demonstrate an important property of engrams – for large forgetting rates the system quickly attains a stationary state at which its “memory” capacity (quantified by the NDC) drops and stays relatively small over time. Interestingly, the steady state memory capacity fluctuates around a quantity (200-300 and 50-100 in terms of NDC for our setup for larger $\alpha$ i.e. $\alpha = 0.02$ and $\alpha = 0.2$ respectively) which is seemingly independent of the characteristic length $l_0$. Hence, the proposed model of the engrams predicts the existence of an intrinsic steady state, which is an important property of learning. Moreover the model with the learning from scratch demonstrates qualitatively similar behavior, as the basic model. For more plots characterizing the kinetics of learning from scratch, for different kinetic parameters see SM, Fig. S1.

### Kinetics of engrams in *d*-dimensional ambient space

#### General setup

Now we consider kinetics of learning in multidimensional systems, in *d*-dimensional ($d> 2$) ambient space. This implies that conceptual space is $d-1$ dimensional, represented by the surface of an appropriate $S^{d-1}$ sphere. In order to compare engrams’ properties for different dimensions *d*, we consider engrams’ dynamics in normalized conceptual space. That is if $(a,b,c,\ldots )$ are features of the stimuli then suppose that $\sqrt{a^2+b^2+c^2 +...}=1$. Based on the nature of the system, we require the following properties of the $d-1$-dimensional normalized manifold: (i) it should not have any boundaries, as the boundaries correspond to a sub-manifold with special properties and (ii) the manifold should be topologically smooth. The surface of a *d*-dimensional ball corresponds to the $d-1$-dimensional manifold with the requested properties. Note that we tacitly assume the homogeneity of the concept space, although concepts themselves may cluster (semantic clustering), possess learning history, etc. However, in a lack of a sufficient knowledge about such effects, we apply a simple *idealized* model of uniform conceptual space—a manifold in an abstract space, where the objects—concepts may be distributed non-uniformly. The same assumption of homogeneity (and for the same reasons) is applied for the space distribution of the hits.

Let us now consider this *d*-dimensional model describing the growth/shrinkage of segments distributed on the surface $S^{d-1}$ of the unit ball $B_{d}(0,1)$ centered at the origin in more detail. The model consists of *n* spherical caps (corresponding to segments in our previous model) $C_{r_{i},\vec {c}_{i}} \subset S^{d-1}$ $(i = 1,2,...,n)$ with distinct radii $r_{i}$, centers $\vec {c}_{i} = (x_{1}, x_{2}, x_{3},..., x_{d})$ ($|\vec {c}_{i}| = 1$) and area $A_{i} \in (0,A_{max}]$. The caps $C_{r_{i},\vec {c}_{i}}$ are randomly distributed on the surface $S^{d-1} \subset B_{d}(0,1)$. Their area is:

10 $$\begin{aligned} A_{i} = \text {area}_{d-1}(C_{r_{i},\vec {c}_{i}})&= \frac{2 \pi ^{\frac{d-1}{2}}}{\Gamma (\frac{d-1}{2})} \int _{0}^{\hat{r}_{i}} \sin ^{d-2} \theta \; d \theta = \frac{\pi ^{\frac{d-1}{2}}}{\Gamma (\frac{d-1}{2})} B\bigg (\sin ^{2}(\hat{r}_{i}),\frac{d-1}{2},\frac{1}{2}\bigg ), \end{aligned}$$

where $\hat{r}_{i}=r_i/R =r_i$ is the polar angle of the cap, with $R=1$ being the radius of the unit ball $B_{d}(0,1)$, and *B*(*x*, *a*, *b*) is the incomplete beta function expressed as

$$\begin{aligned} B(x,a,b) = \int _{0}^{x} t^{a-1} (1-t)^{b-1} dt. \end{aligned}$$

For instance, in the dimension $d = 3$, the cap $C_{r_{i},\vec {c}_{i}} \subset S^{2}$ centered at the point $\vec {c}_{i} = (x_{1},x_{2},x_{3})$ and located on the surface of the unit sphere $B_{3}(0,1)$ will have the following area:

$$\begin{aligned} A_{i} = 2 \pi (1 - \cos (\hat{r}_{i})). \end{aligned}$$

The maximum area the spherical cap is allowed to grow to is denoted by $A_{max}$.

As we have seen in the previous section, the confirming learning and learning from scratch both possess steady-state, whose properties do not differ much. Therefore, we will focus below on the confirming learning. Similar to the case of 1 dimensional model studied which we discussed and analyzed earlier, catching shots successively drop onto $S^{d-1}$, at the rate $1/\tau$, striking points $\vec {X}_{p} = (x_{1}, x_{2},..., x_{d})$. They may hit none, one, or several spherical caps at a time. The caps are allowed to overlap. As before, the shots instantly evaporate when none of the spherical caps get hit. This corresponds to a state in the model when the stimulus is completely missed and left “no impression” on the existing engrams. When a catching shot falls inside $C_{r_{i},\vec {c}_{i}}$, the center $\vec {c}_{i}$ of the hit spherical cap moves to the point of the catching shot $\vec {X}_{p}$. The area of the displaced cap then reduces in accordance with the Poisson distribution with the average shrinkage size of $A_{0}$. If a cap is not hit, then its area $A_i$ grows at the rate $\alpha e^{-A_i/A_{\max }}\Theta (A_{\max }-A_i)$.

The engram kinetic equation for *d*-dimensional case is a straightforward generalization of the respective one-dimensional equation:

11 $$\begin{aligned} \frac{\partial f(A,t)}{\partial t}&= - \frac{A}{\tau A_{*}} f(A,t) - \frac{\partial }{\partial A} \bigg (\frac{dA}{d t}\bigg ) f(A,t) + P_\textrm{foc}(A) \int _{0}^{A_{max}} f(A',t) \frac{A' }{A_{*}\tau } dA', \end{aligned}$$

where *A* is the size ( $d-1$-dimensional area of an engram), $A_*$ – the total area of the conceptual space. We assume that the forgetting (that is, the increase of an engram area) and focusing after a conceptual hit (that is, an engram shrinking after a hit) occur in a similar way as in one-dimensional case.

Hence, *d*-dimensional *kinetic equation for engrams* may be derived in the same way as for one-dimensional model:

12 $$\begin{aligned} \frac{\partial f(A,t)}{\partial t}&= - \frac{A}{\tau A_{*}} f(A,t) - \frac{\partial }{\partial A} \bigg (\alpha e^{-A/A_\textrm{max}} \Theta (A_\textrm{max}-A)\bigg ) f(A,t) + A_0^{-1}e^{-A/A_0} \int _{0}^{A_{max}} f(A',t) \frac{A' }{A_{*}\tau } dA'. \end{aligned}$$

Here *A* denotes of the area (size) of the engram, $A_{*}=2 \pi ^{d/2}/\Gamma (d/2)$ is the surface area of the corresponding unit sphere in *d* dimensional ambient space, and

13 $$\begin{aligned} P_\textrm{foc}(A)= & A_0^{-1}e^{-A/A_0} \end{aligned}$$

14 $$\begin{aligned} \frac{dA}{dt}= & \alpha e^{-A/A_\textrm{max}} \Theta (A_\textrm{max}-A). \end{aligned}$$

For the simplified model discussed in Sec. 2.1, and which is more rigid in terms of phenomenological plausibility and mathematically less convenient due to singularities at $A=A_0$, one could choose $P_\textrm{foc}(A)= \delta (A-A_0)$ and $dA/dt = \alpha A \Theta (A_\textrm{max}-A)$. In the above equations, Eqs. (13), (14), we assume that the conceptual space is uniform, so that *f*(*A*, *t*) does not depend on the coordinates $\vec {X}=(x_1,x_2, \ldots , x_d)$. The structure of Eqs. (12)–(14) is identical to Eq. (6). Therefore, the overall description of the steady-state solution of these equations coincides with Eq. (9), up to an appropriate change of notation. Note that kinetic equations (6) and (12)–(14) are essentially mean-field equations, which lack fluctuations.

To study kinetic properties of the model, reflecting fluctuations, we conducted relevant Monte Carlo simulations, associated with the process (see Methods and Supplementary Material (SM) for detail). For each dimension, $n = 1000$ spherical caps are randomly distributed on the surface $S^{d-1}$ of the unit ball $B_{d}(0,1)$. Two kinetic parameters are investigated: the growth rate $\alpha$ (the forgetting rate) and counterpart—the expected focusing/shrinkage size $A_{0}$ (the “focusing” parameter).

The aim is to investigate the impact of $\alpha$ and $A_{0}$ on the number of distinct centers (NDC) at large terminal times *t* corresponding to the end time of the simulation. Recall that our model enables multiple engrams to collide and share the same centres as a result of the collision. Exploring the entire two-dimensional parametric space is computationally challenging for large *t*. Therefore, we assumed that the forgetting rate and the focusing are correlated. In particular, we confined our exploration to the case when smaller forgetting rates imply that the focusing is sharper and vice-versa. We explored the evolution of engrams at the following values of $\alpha$ and $A_0$: $\alpha = 2 \times 10^{-p}$ and $A_{0} = 1 \times 10^{-p}$, where *p* is a positive constant which we varied from one experiment to another. This enabled us to assess the system’s behavior across a broad range of temporal and spatial scales. (In practice, since $A_{max}(d)$ decreases with *d* and becomes small, we need to use the values of *p* satisfying the condition $A_{0} < A_{max}(d)$, which limits *p* from below.)

The rate of arrival of the catching shots was set to one. This fixed the time unit of the kinetic process. By varying the values of $\alpha$ we could probe different engram evolution regimes. This included the regime when spherical caps grow rapidly after being hit but the frequency of arriving shots is low, $\alpha \gg l_0/\tau$. This regime corresponds to a system of “fast forgetting”. We also looked at the regime when the growth of caps is slow relative to the frequency of arriving shots, $\alpha \ll l_0/\tau$, which could be interpreted as a system with “slow forgetting”. The value of $A_{max}$ was set to $\pi ^{d/2}/2\Gamma (d/2)$ (quarter of the size of the conceptual space) in all modeling scenarios.

#### Simulation results for *d*-dimensional ambient space

In our MC simulations we explored the evolution of engrams for $d\ge 3$. The main macroscopic feature we tracked in our simulations was the dynamics of the number of distinct centers over time and the number of distinct centers at the end of the temporal evolution of the system as a function of dimension *d*.

**Fig. 4:**
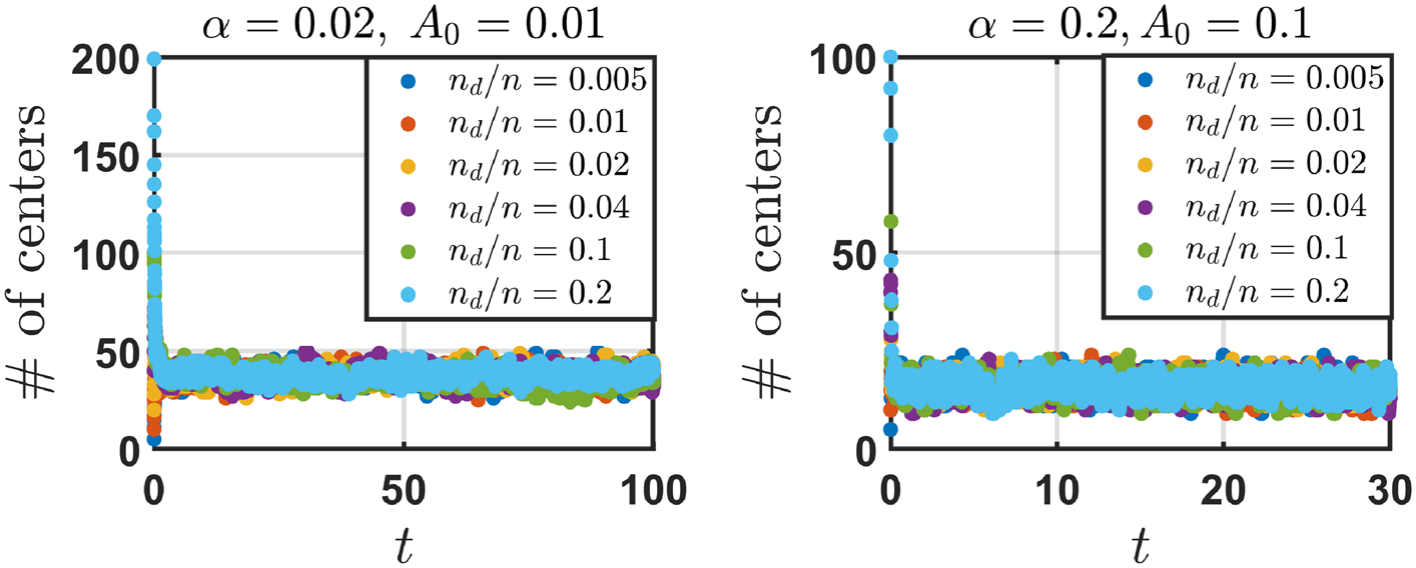
The number of distinct engram centers (NDC) over time *t* in 3d case, for parameters $(\alpha , A_{0},d) = (0.02,0.01,3)$ (left) and $(\alpha , A_{0},d) = (0.2,0.1,3)$ (right). Different initial number of distinct engram centers $n_{d}$ are investigated with $n = 1000$ total number of engrams. The ratio $n_{d}/n$ determines whether NDC monotonically/non-monotonically increases/decreases over time. Eventually, the number of engrams with different centers in the conceptual space (NDC) over time fluctuates around the same steady state value for any initial number of distinct engram centers.

We observe that the number of distinct engram centers (NDC), after a short transient period, converges to a vicinity of some steady state value and keeps fluctuating around this state for the remaining time of the simulation. This initial increase/decrease in the number of distinct engram centers is mostly dependent on the ratio of the initial number of distinct centers $n_{d}$ and the total number of engrams *n*. When the ratio $n_{d}/n$ is small at the start of the evolution, engrams sharing a common centre but whose sizes are different break apart over time. This results in an increase in the number of distinct engram centers over time before the steady state is reached. The opposite occurs when $n_{d}/n$ is sufficiently large. NDC decreases as engrams continuously group together over time. Numerical values of the steady state did not visibly change for different ratios $n_{d}/n$ (as seen in Fig. 4). That is, the steady state value of engrams with different centers was largely independent on the initial number of engrams with distinct centers. When the areas of the hit engrams shrink to the same value under the Poisson distribution, engrams in the same groupings do not break apart.

**Fig. 5:**
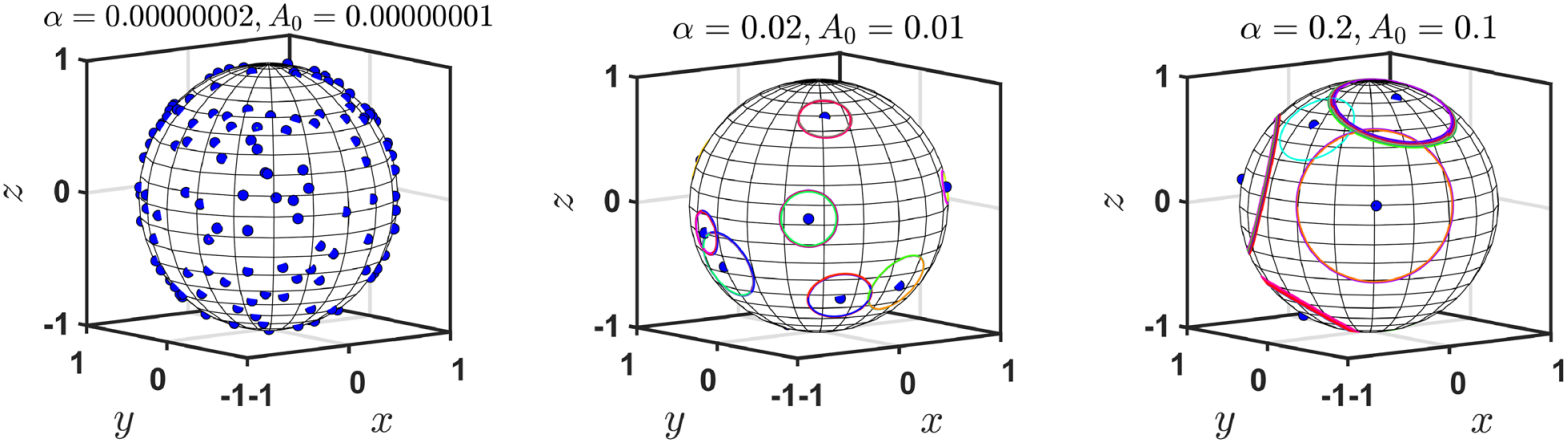
The parameters $\alpha$ and $A_{0}$ significantly affect the steady state distribution of engrams. In this 3d example, we observe a large number of small-size individual engrams when $(\alpha ,A_{0})$ are small (left) and a small number of large individual engrams when $(\alpha ,A_{0})$ are sufficiently large (right). The former case a large part of the conceptual space remains uncovered by the engrams, while in the latter case it is much more covered. This figure was produced using Matlab R2022b.

Figure (5) visualizes the model engram distribution in the steady state depending on the kinetic parameters of the model, $(\alpha ,A_{0})$. When $\alpha$ and $A_{0}$ are both large, there is a small number of independent engrams with different centers and a large number of engrams sharing the same center—they form a nested system of engrams. Contrary, when $\alpha$ and $A_{0}$ are both small, the model predicts a large number of small independent engrams. Hence one can clearly see two opposite tendencies—large forgetting rate (large $\alpha$), with less focusing rate (large $A_0$) result in larger covering of the conceptual space by the engrams, but they are less focused and many engrams share the same center (i.e. the dominance of nested engrams is observed). In contrast, for small forgetting rate and sharp focusing, all engrams are more focused and specialized (only a few share the center), but the total conceptual space is significantly less covered by the engrams. Possibly, there exist optimal values of $(\alpha ,A_{0})$ which correspond to the trade-off between these two tendencies.

#### Critical dimension for evolving engrams

**Fig. 6:**
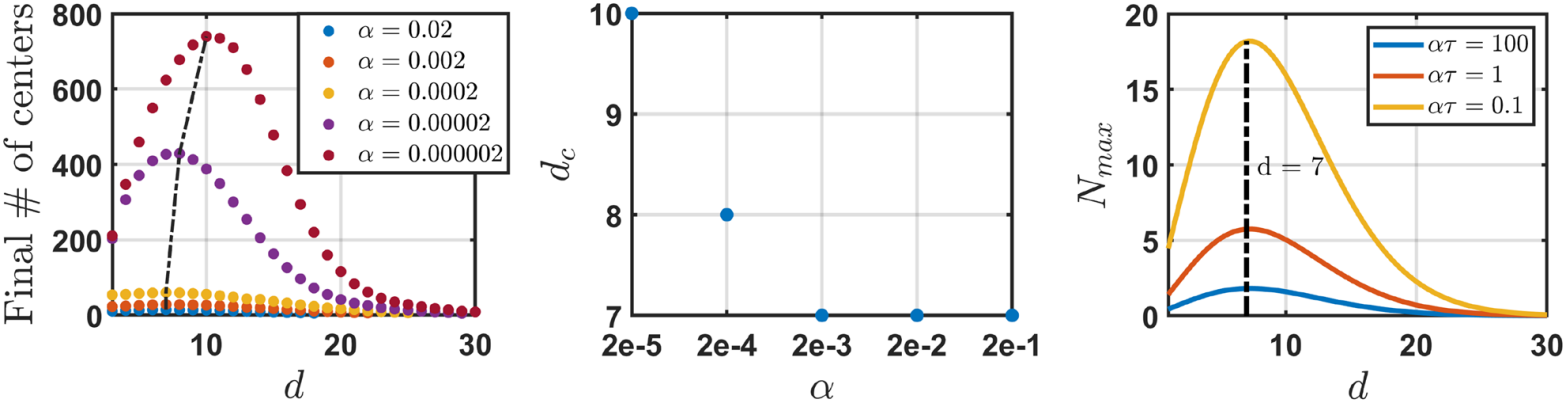
Left panel: The steady state number of different engram centers (NDC), as the function of *d*—the dimension of the conceptual space. A critical dimension $d_c$ exists where the number of different engrams is maximal. For $d > d_{c}$ the number of different centers decreases with dimension tending to one as $d \rightarrow \infty$. Middle panel: The critical dimension $d_c$ as a function of the forgetting rate $\alpha$. $d_c$ decreases with increasing $\alpha$ and saturates at $d_c = 7$. Right panel: The maximal number of distinct engrams as a function of the dimension of the ambient space, as prescribed by Eq. (18).

Next, we explore the dependence of the number of separate engrams in the steady state on the dimension of the ambient space *d*; the results are presented in Fig. 6. Surprisingly, this dependence is not monotonous. Namely, the number of different engram centers initially increases with *d*, reaching a maximum and then decreases, see Fig. 6 (left). Interestingly, the critical dimension slightly decreases when the forgetting rate $\alpha$ increases. That is, the smaller the forgetting rate the larger the critical dimension. At the same time, we observed that for larger values of the forgetting rate $\alpha$ critical dimension $d_s$ saturates around $d_c=7$ for $\alpha > 10^{-4}$, see Fig. 6 (right). This suggests that the region around $d_c=7$ may correspond to an optimal dimension in terms of the capacity of the conceptual space.

Below we present a qualitative theoretical explanation for the existence of the critical dimension. Let $A_{ tot}(t)$ be the total area occupied by *N*(*t*) engrams with different centers at some time instance *t* in *d*-dimensional ambient space. Then the average area occupied by a single engram is $A_{ tot}(t)/N(t)$. Suppose that $A_{ tot}(t)/N(t)>A_0$. When an engram is hit by a catching shot, its area, on average, shrinks to $A_0$ (see see Eq. (13)). Let *m* be the average number of (overlapping) engrams affected by a single hit. Then each hit, on average, reduces the total area occupied by engrams by $m(A_{ tot}(t)/N(t) - A_0)$. Given that the average hit rate can be estimated as $1/\tau \cdot A_{tot}/A^*$, where $1/\tau$ is the relative frequency of shots and $A^*$ is the area of the entire concept space, the overall reduction of the area covered by engrams due to the system’s exposure to stimuli can be estimated as:

$$\frac{A_{tot}(t)}{\tau A^*}m(A_{ tot}(t)/N(t) - A_0).$$

At the same time, the growth rate of the total area occupied by *N* engrams with different centers, may be approximated by the growth rate of a single engram of the average size $A_{tot}/N$, which is $\alpha e^{-A_{tot}/(N\, A_{max})}$, multiplied by the number of engrams *N*, yielding

$$N \alpha e^{-A_{tot}/(N\, A_{max})},$$

where we neglect that engrams may overlap and skip the factor $\Theta (A_{max}-A_{tot}/N)$, see the discussion below. Hence, one can write the following mean-field kinetic equation governing the dynamics of the total area occupied by engrams:

15 $$\begin{aligned} \frac{d}{dt} A_{ tot} \simeq \alpha N \,e^{-A_{tot}/(N\,A_\textrm{max})} - \frac{A_{ tot}}{\tau A_*} \left[ m \left( \frac{A_{ tot}}{N} - A_0 \right) \right] . \end{aligned}$$

For a steady state, $dA_{tot}/dt=0$, one obtains,

16 $$\begin{aligned} \alpha N \,e^{-A_{tot}/(N\,A_\textrm{max})} \simeq \frac{A_{ tot}}{\tau A_*} \left[ m \left( \frac{A_{ tot}}{N} - A_0 \right) \right] . \end{aligned}$$

We assume, for simplicity, that $A_0$ is very small, $A_0 \ll A_{tot}/N$, while $A_\textrm{max}$ is large, $A_\textrm{max} \gg A_{tot}/N$. In a steady state, $dN/dt=0$ and the engrams do not overlap. This is because any hit in the overlapping area decreases the number of distinct centres *N*. Therefore, $m=1$, and Eq. (16) implies that the following relationship holds true in the steady state:

17 $$\begin{aligned} N^2 \approx \frac{A_{ tot}^2}{\alpha \tau A_* } . \end{aligned}$$

Let us estimate the maximal number of distinct engrams. In this case, the total coverage is close to maximal, since otherwise there is a room for an additional engram in the unoccupied area, which contradicts to the assumption that the number of engrams is maximal. Therefore $A_{tot} \simeq A_* =2 \pi ^{d/2}/\Gamma (d/2)$, which finally yields

18 $$\begin{aligned} N_\textrm{max}(d) \simeq \sqrt{ \frac{2 \pi ^{d/2}}{\alpha \tau \, \Gamma (d/2)}} . \end{aligned}$$

The dependence $N_\textrm{max} (d)$, given by Eq. (18) is shown in Fig. 6 (Right panel). As it may be seen from the figure, the qualitative theory adequately predicts the critical dimension observed in experiments. Strictly speaking, one should use the relation, $A_{tot}=A_*\varphi _d$, where $\varphi _d =\varphi \left( d, f(A)\right)$ is the “packing fraction”, which depends on the dimension and on the distribution of engrams’ areas *f*(*A*). The latter has a form that is similar to the one shown in Fig. 2, with a broad distribution of engrams’ areas—from very small values to very large ones. For the 3-d systems, it is known that the packing fraction tends to 1 (the void fraction vanishes) with increasing dispersion of spheres’ radii, see e.g.^35,36^. Therefore we use here a reasonable conjecture, that $\varphi _d \approx 1$, since the distribution *f*(*A*) demonstrates a large dispersion of engrams’ areas with the abundance of small-size engrams.

Note, that the derivation of Eq. (18) for the maximal number of independent engrams is based on the kinetic equation (15) for the total areas of all engrams. Therefore, Eq. (15), and hence Eq. (18), remain valid for the case of non-uniform distribution of hits over the conceptual space and space-correlated stimuli, since it deals with $A_{tot}$—the integral property which is not sensitive to reasonable fluctuations in spatial characteristics of engrams’ distribution. One can also consider the case of time-correlated stimuli that will also yield (18). In the SM we demonstrate that all the above cases—with space non-uniform stimuli, space and time-correlated stimuli result in the same Eq. (18), but with the renormalized hit rates $\tau _*$.

#### Kinetics of engrams with inhibition

Let us consider now somewhat more detailed model for evolution of an engram group, when a conceptual hit acts on intersecting engrams in the conceptual space. The engrams differ by the distance of their centers from the hit point in the conceptual space. Then we consider two models, associated with the (i) closest distance between the engram center and hit point and (ii) with the largest distance. For both models the centers of all hit engrams move to the hit point. However, for model, (i), only the engram with the shortest distance shrinks, according to the Poisson distribution, while for the second model, (ii), only the engram with the longest distance shrinks. We call this model engrams with inhibition.

**Fig. 7:**
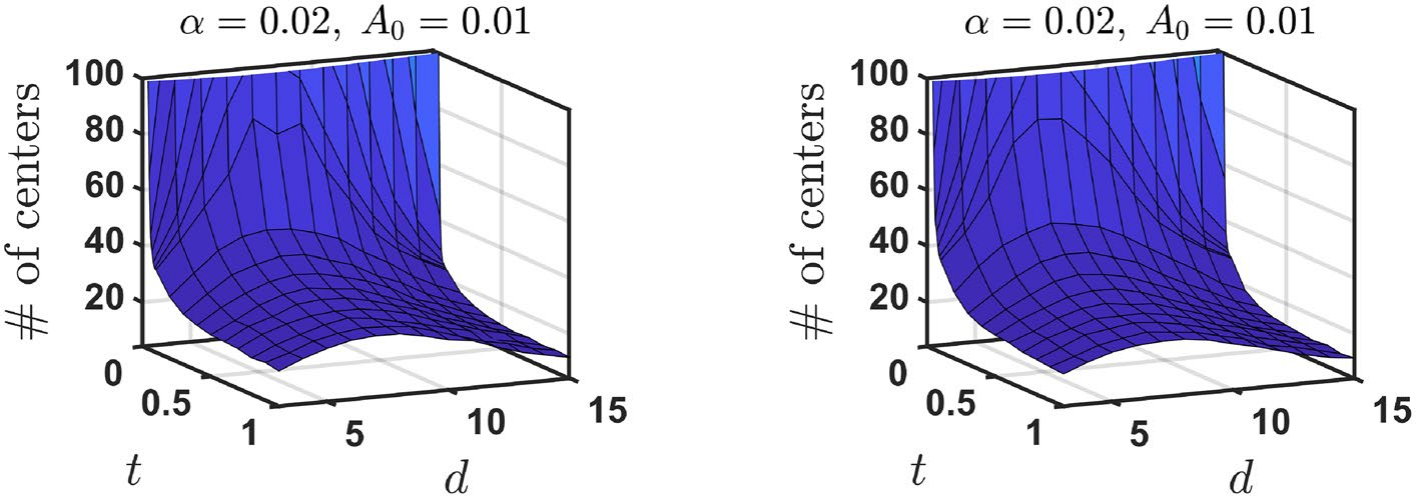
The number of engrams with different centers (NDC) over dimension *d* for different time, for two models with inhibition. Left panel—the model (i), when only engram with the minimum distance of its center from the hit point shrinks. Right panel—the model (ii), when only engram with the largest distance of its center from the hit point shrinks (see the text for more detail). The maximum area $A_{max}$ is $A_{max} = (\pi ^{d/2}/2\Gamma (d/2))$. Matlab R2022b was utilized to generate this figure.

Similarly to the basic model we performed MC simulation for the kinetic model with inhibition, see Supplementary Materials for detail. We observe the same qualitative behaviour as found for the basic model. At small time *t*, number of engrams with different centers increases with dimension, but with growing time it becomes non-monotonic function of *d*, as it is illustrated in Fig. 7. Furthermore, for small focusing constant $A_0$ the basic model and model with inhibition predict different steady state number of engrams, although for large $A_0$ steady states for basic and two models with inhibition coincide; the behavior of the average overlapping area with time is also similar to this of the basic model, see Supplementary Materials.

## Methods

In our numerical experiments, we used Monte Carlo simulations to produce trajectories of relevant variables characterising memory engrams. Algorithm 1 presents numerical workflow for the basic model. Algorithm 2 describes the process we used to simulate the kinetic model with inhibition and Algorithm 3 describes the Monte Carlo simulation steps for the one-dimensional process of learning from scratch.

**Algorithm 1:**
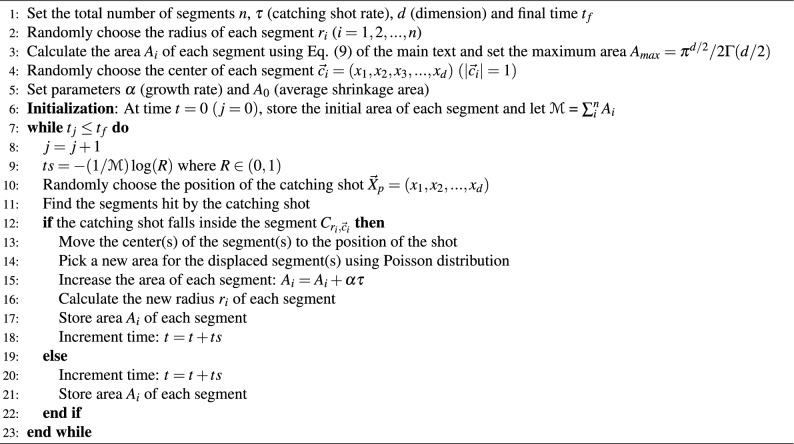
Monte Carlo Simulation for *d*-dimensional model

**Algorithm 2:**
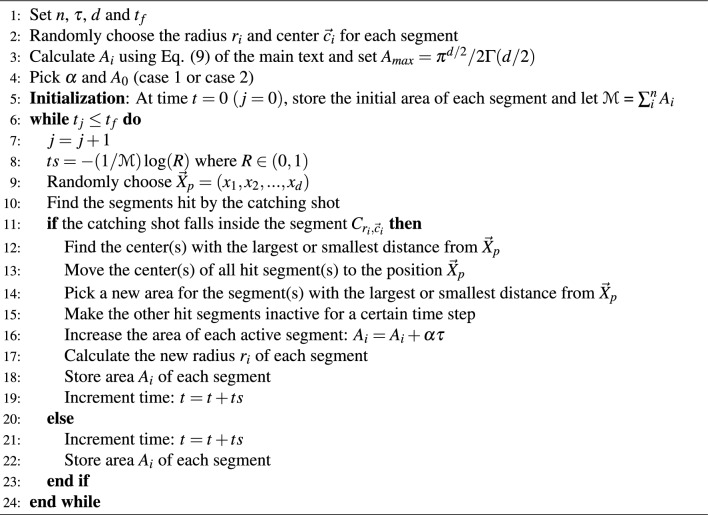
Monte Carlo Simulation for *d*-dimensional model with inhibition

**Algorithm 3:**
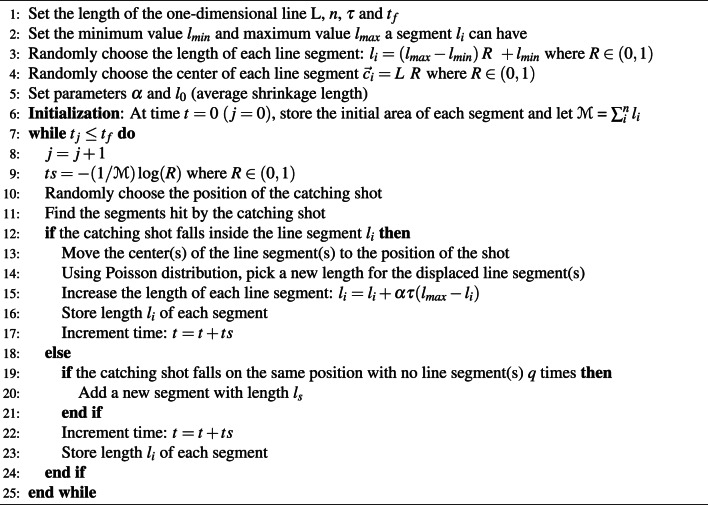
Monte Carlo Simulation for the one-dimensional model for Learning from scratch

## Discussion and conclusion

This work introduces kinetic models of memory engrams. Engrams in our models are represented by evolving geometrical objects in *d*-dimensional conceptual space. These objects are not rigid as several engrams can intersect, partially occupying the same region in the conceptual space, or can even form nested structures, when one engram resides inside a bigger one. The engrams respond to particular “impressions”—stimuli from the external world, which hit points of the conceptual space. If a stimulus hits an engram, the latter responds, otherwise the hit does not change the conceptual space. The stimuli hitting the engrams change their size and location. This mimics the learning process in terms of engrams. The action of a stimulus (hit) onto an engram has two consequences: (i) it confirms the corresponding concept and rectifies it, and (ii) it makes the concept more focused. The first action (i) is modelled by the shift of the geometric center on an engram to the point of the hit. The second action (ii) is modelled by the shrinkage of the engram to smaller objects in the conceptual space. In between stimuli engram permanently and steadily enlarge. This process mimics the forgetting of a concept—the engram becomes less and less focused. Hence in our model we introduce a set of kinetic coefficients, characterising the forgetting rate and the focusing rate of learning.

Based on this simple geometric model of memory and learning by engrams, we derived a kinetic equation for the characteristic function of engrams which describes the engram size distribution in the conceptual space. We start from a toy-model of one-dimensional conceptual space and find an analytical steady-state solution of the *engram kinetic equation.* The analytical result is in an excellent agreement with the respective numerical solution of the engram equation. Then we consider *d*-dimensional conceptual space and solve the engram kinetic equation by means of Monte Carlo approach. We analysed the time evolution of the engram characteristic function and observe that it tends, in the course of time, to a steady state distribution. Both evolution of the function and its steady state sensitively depend on the kinetic coefficients of the model—on the forgetting rate $\alpha$ and focusing constant $A_0$. We analyze in detail such an important characteristic of the engram ensemble, as the number of different centers of engrams in conceptual space. We observe that this quantity is not a monotone function of dimension of the ambient space. Instead, it peaks at an extremal dimension $d_c$. The value of $d_c$ decreases with the forgetting rate $\alpha$ and saturates at $d_c=7$ for larger values of $\alpha$.

To understand the phenomenon of critical dimension, we developed a mean-field theory of the evolution of engrams. The new theory offers an explanation for the existence of critical dimension. Moreover, it gave a correct quantitative prediction of the value of the critical dimension ($d_c=7$). Through numerical simulations, we found that for small forgetting rate and strong focusing, the number of engrams with different centers is large, while the size of engrams in the steady state is relatively small. This corresponds to the sharpening of concepts—all engrams become very focused. At the same time, a significant part of the conceptual space remains uncovered. This means that a large fraction of stimuli remains unnoticed by the system, which corresponds to a relatively poor learning. At the opposite end of the spectrum, when forgetting rate is large and focusing is weak, the engrams are relatively large and the uncovered area of the conceptual space is relatively small. This corresponds to much more effective learning, when most of the stimuli cause changes in the conceptual space. However, the learned concepts are not sharp and many engrams overlap. Hence in the studied engram model of learning we observe an inherent trade-off between the receptivity to new stimuli (“readiness for learning”) and sharp selectivity of learning. This is reminiscent of classical bias-variance trade-off in statistical learning theory^37^.

In addition to revealing and justifying the existence of critical dimension in the space of concepts, the proposed kinetic model of the dynamics of memory engrams may offer novel interpretation of empirical data, both existing and new. For example, one can empirically examine and explore the mechanisms of engrams’ merging and fragmentation. Suppose two different yet sufficiently close (in conceptual space) engrams are formed in a subject. Then, at later time, the same subject receives a new stimulus (an impression) which could be associated with either of the two engrams. The research question would be to investigate the probability of merging these two concepts into a single one as a function of both timing between the events and intensity of stimulation. Depending on the outcomes (preferential sharpening of one engram over the other, merging, or dissociation of engrams) we would then be able to verify a particular regime of engrams’ response and interaction. One can also imagine a hypothetical experiment when a subject is exposed to an external stimulus (a new experience which could be viewed as a precursor of a memory concept) at a time $t_0$. If this stimulus is sufficiently strong/important then we may assume that it will produce a sharpened reflection in the subject’s mind corresponding to a concept. After some time, at $t>t_0$ in the same experiment, we expose the subject to another stimulus. After some time, we can then ask the subject report if the second stimulus was detected or not. The minimal value of $t-t_0$ corresponding to the pairs of stimuli which the subject was able to detect could server as an estimate of the value of $\tau$ in our model.

In addition to basic kinetic model of evolving engrams corresponding to a system with pre-existing engrams (confirming learning), we also studied two related models: learning from scratch and kinetic engrams with inhibition. In the former model, new engrams emerge when an impression hits the same area of the conceptual space several times in a row. In the latter model, we assume that when an impression hits overlapping engrams, only one engram whose center is the closest to the location of the hit experiences focusing shrinkage. We have found that all the studied extensions of the basic model have qualitatively similar behavior as the basic kinetic engram model. One of the intriguing consequences of the model is the apparent existence of the optimal number of senses in evolving neural and neuromorphic systems. Indeed, suppose that the number of features associated with stimuli has a one-to-one correspondence with the number of human or artificial agent’s senses. Then the dimension of engrams in the conceptual space coincides with the number of senses. In this case, the largest capacity of the conceptual space, that is, the most rich perception of the external world, would be attained when the number of senses is equal to 7 - the critical dimension where the number of survived/retained different concepts is maximal.

To conclude, we believe that out kinetic engram model, based on the geometric representation of engrams in conceptual *d*-dimensional space will allow to understand qualitatively basic properties of learning and memory.

## Supplementary Information

Supplementary Information.

## Data Availability

The datasets used and/or analysed as a part of the current study are available from the corresponding author upon reasonable request.
