# Supplementary material for: The critical dimension of memory engrams and an optimal number of senses
Source: Sci Rep. 2025 Aug 15;15:29972. doi: 10.1038/s41598-025-11244-y (PMC12356968; doi:10.1038/s41598-025-11244-y)
Supplement: Supplementary file 1 — Supplementary Information. [file 41598_2025_11244_MOESM1_ESM.pdf]

Supplementary Material for The critical dimension of memory engrams and an optimal number of senses

Wendy Otieno[1], Ivan Y. Tyukin[2,3,*], and Nikolay Brilliantov[3,4]

[1]Department of Physics, Loughborough University, Loughborough LE11 3TU, United Kingdom
[2]King's College London, London, Strand, WC2R 2LS, United Kingdom
[3]Skolkovo Institute of Science and Technology, Bol'shoi Bulvar 30, Moscow, 121205, Russia
[4]University of Leicester, University Road, Leicester, LE1 7RH, United Kingdom
[*]ivan.tyukin@kcl.ac.uk

Learning from scratch

Below we present more plots for the learning from scratch (see Fig S1), which supplements the data presented in the main text.

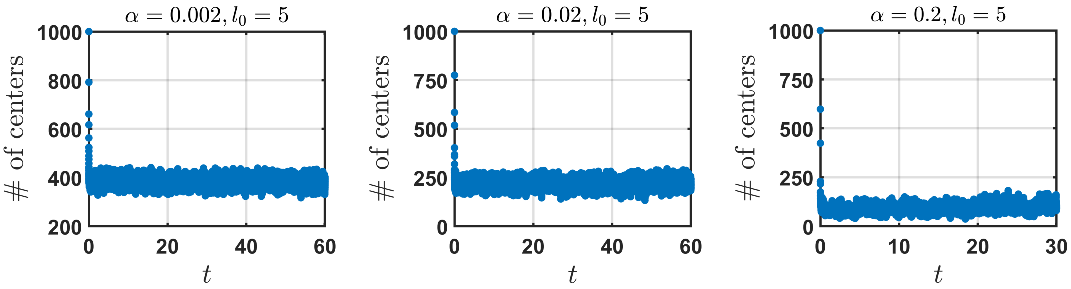

Figure S1. Number of distinct centers (NDC) over time for different α. After some transient time NDC reaches a steady state.

Evolution of the average overlapping area

Basic model

The normalized average overlapping area (AOA) can exhibit one of three main behaviour: (1) it can decrease with time and saturate at a finite value close to zero (2) it may have irregular cycles of increasing and decreasing AOA or 3) it may experience strong fluctuations. For dimension $d = 3$, the normalized AOA initially decreases with time (see Fig S2). For relatively large α and A_0 (small exponent p), AOA decreases with time and approaches a value that is close to zero. This is because hit segments shrink to a small area relative to A_{max} and the area of all segments also increase to an area that is minuscule. This leads to segments overlapping less as time goes by to the point where the probability of hitting segments is small. As A_0 increases, the normalized AOA starts experiencing irregular cycles of AOA increasing and decreasing. These irregular cycles at latter times turn into strong fluctuations when α and A_0 are small (the exponent p is large). The same qualitative behaviour occur for dimension $d = 8$ (see middle column plots). For sufficiently large dimensions d, the normalized AOA has irregular cycles of increasing and decreasing for large α and A_0 and strong fluctuations at small α and A_0.

Model with inhibition

In Fig. S3 the number of engrams with different centers as a function of time for different dimensions is shown. As one can see from the figure, for small focusing constant A_0 the basic model and model with inhibition predict different steady state number of engrams, while for large A_0 the number of engrams steady states coincide for the basic and both models with inhibition. The behavior of the average overlapping area with time is also similar to that of the basic model. That is, the normalized average overlapping area (AOA) decreases with time and exhibit an irregular cycle of increasing and decreasing for the minimum and maximum distance case (see Fig S4). For the smallest dimension d, the amplitude of the increase and drop is minuscule. This is because of the average shrinkage area A_0 being small along with a small growth rate α. As dimension increases, so does the amplitude. In dimension 16, we find that at earlier times all the cases have large amplitudes but at latter times the amplitude of the irregular cycles decreases.

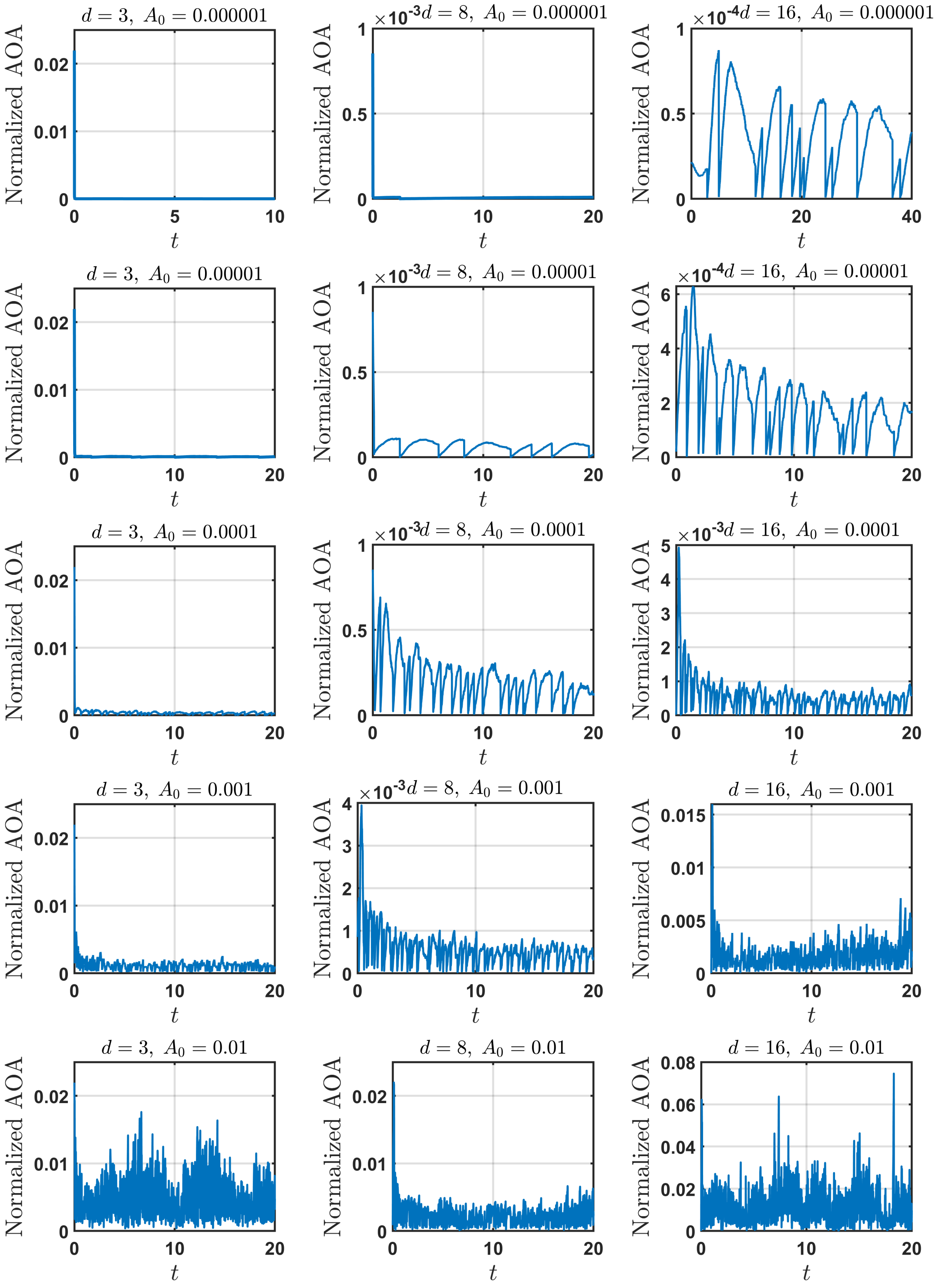

Figure S2. Normalized average overlapping area (AOA) – the average overlapping area over the surface area of the d-th dimensional unit sphere, for parameters $(\alpha, A_0) = (2\times 10^{-p}, 1\times 10^{-p})$ where $p = 2$, 3, 4, 5 and 6 for the dimensions $d = 3$ (left column), $d = 8$ (middle column) and $d = 16$ (right column). For small exponent p, the normalized AOA decreases with time and approaches zero at dimensions $d = 3$ and $d = 8$. At $d = 16$, the normalized AOA increases and then proceeds to fall irregularly (see top plots). For large exponent p, the normalized AOA strongly fluctuates for all dimensions (see bottom plots).

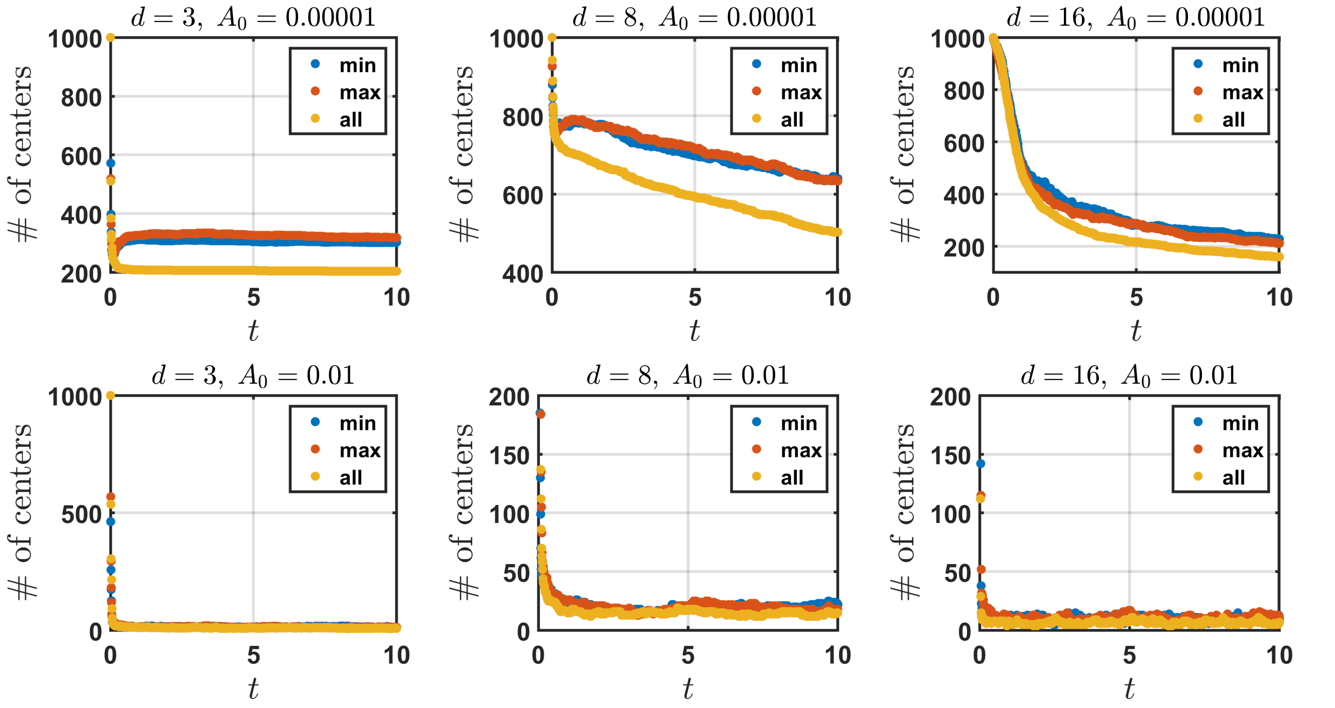

Figure S3. The number of engrams with different centers as a function of time for $d = 3$ (left panels), $d = 8$ (middle panels) and $d = 16$ (right panels) for $A_0 = 10^{-5}$ (upper panels) and $A = 0.01$ (bottom panels). Here the notation "min" is associated with the inhibition model, when only the engram with the shortest distance, between its center and hit point, shrinks, according to the Poisson distribution. "Max" corresponds to the inhibition model, where only the engram with the longest distance between its center and hit point shrinks. "All" refers to the basic model, when both hit engrams shrink.

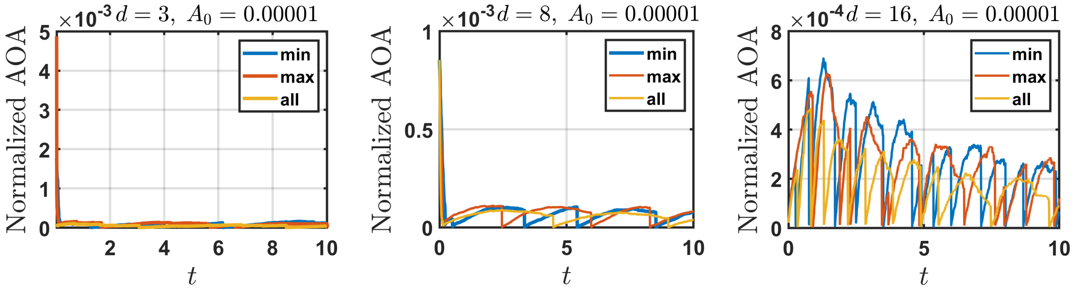

Figure S4. Normalized average overlapping area over time for $d = 3$, $d = 8$ and $d = 16$. NAOA decreases with time and experiences an irregular cycle of increasing and dropping for the minimum and maximum distance case. At large dimension $d = 16$, all the cases have large amplitudes at earlier times. The notations are the same as in Fig. S3.

For large A_0 and α, the number of unique centers decreases with time for all cases and their quantities are close for all dimensions. AOA experiences large fluctuations for all dimensions d (see Fig S5).

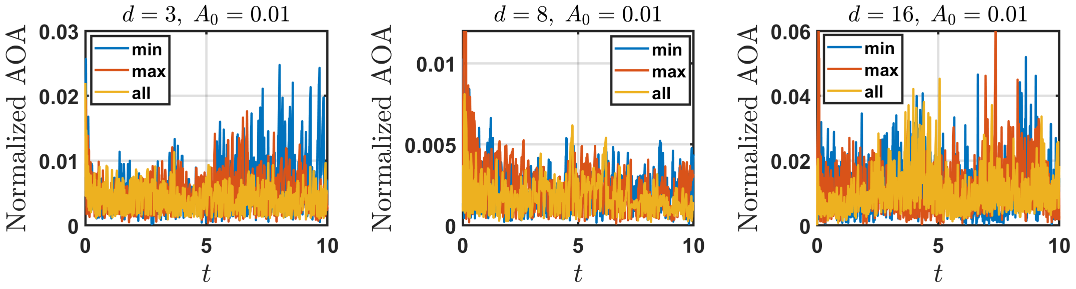

Figure S5. Normalized average overlapping area over time for $d = 3$, $d = 8$ and $d = 16$. The normalized AOA experiences large fluctuations for all dimensions d. The notations are the same as in Fig. S3.

Steady state distribution function of engrams for the simplified model

For the oversimplified model with $P_{\text{foc}}(l) = \delta(l - l_0)$ and $dl/dt = \alpha\,\Theta(l_{max} - l)$ we obtain the following kinetic equation instead of Eq. (6) of the main text:

$$\frac{\partial f(l,t)}{\partial t} = -\frac{l}{\tau l_*} f(l,t) - \alpha \frac{\partial}{\partial l}\left[\Theta(l_{max} - l) f(l,t)\right] + \frac{F(t)}{\tau l_0}\delta(l - l_0). \tag{1}$$

If we neglect the non-physical singularity at $l = l_{max}$ obtained after the differentiation over l, we obtain the following equation for the steady-state distribution $f(l)$:

$$\frac{df}{dl} = -\frac{l}{\alpha\tau l_*} f + \frac{F}{\alpha\tau l_0}\delta(l - l_0). \tag{2}$$

Its general solution is:

$$f(l) = Ce^{-Q(l^2 - l_{min}^2)/2} + e^{-Ql^2/2}\int_{l_{min}}^{l} e^{Qx^2/2}\frac{F}{\alpha\tau l_0}\delta(x - l_0)dx, \tag{3}$$

with $Q = 1/(\alpha\tau l_*)$, and constant C determining the boundary condition at $l = l_{min}$. Since we expect that in the simplified model governed by Eqs. (1), (2), there are no engrams with $l < l_0$ in the steady state, we use $l_{min} = l_0 - \varepsilon$, with $\varepsilon > 0$, $\varepsilon \to 0$, and $f(l_{min}) = 0$. In this case $C = 0$ in (3) and hence

$$f(l) = (F/\alpha\tau l_0)\exp[-(l^2 - l_0^2)/(2\alpha\tau l_*)]\Theta(l - l_0),$$

where $\Theta(x) = 1$ for $x \geq 0$ and $\Theta(x) = 0$ for $x < 0$.

Maximal number of engrams for space inhomogeneous, time and space correlated stimuli

Maximal number of engrams for space inhomogeneous stimuli

Let the frequency of hits is not homogeneous in the concept space, and depends on the coordinate $\mathbf{r}$ there. That is the probability of a hit during the time interval dt at the small area $d\mathbf{r}$ around a point $\mathbf{r}$ reads $dtd\mathbf{r}/\tau(\mathbf{r})A_*$. Hence the probability to hit total occupied area A_{tot} reads

$$\int_{\mathbf{r}\in A_{tot}} \frac{d\mathbf{r}dt}{\tau(\mathbf{r})A_*}.$$

Applying the same reasoning, as in the derivation of the mean-field kinetic equation Eq. (14) of the main text we obtain,

$$\frac{d}{dt}A_{tot} \simeq \alpha N e^{-A_{tot}/(NA_{\max})} - \int_{\mathbf{r}\in A_{tot}} \frac{d\mathbf{r}}{\tau(\mathbf{r})A_*}\left[m\left(\frac{A_{tot}}{N}-A_0\right)\right], \tag{4}$$

with the same meaning of all the terms as in Eq. (14) of the main text. Assuming, as in the main text, that $A_0 \ll A_{tot}/N$, $A_{\max} \gg A_{tot}/N$, we obtain for the steady state, taking into account that in this case $m=1$,

$$N^2 \simeq \frac{A_{tot}}{\alpha A_*}\int_{\mathbf{r}\in A_{tot}} \frac{d\mathbf{r}}{\tau(\mathbf{r})}.$$

Using again that for $N = N_{max}$ the condition $A_{tot} \simeq A_*$ holds true, we find

$$N^2 \simeq \frac{A_*}{\alpha A_*}\int_{\mathbf{r}\in A_*} \frac{d\mathbf{r}}{\tau(\mathbf{r})} = \frac{A_*}{\alpha\tau_*}, \qquad \tau_*^{-1} \equiv \frac{1}{A_*}\int_{A_*} \frac{d\mathbf{r}}{\tau(\mathbf{r})}.$$

With $A_* = 2\pi^{d/2}/\Gamma(d/2)$, we find

$$N_{\max} \simeq \sqrt{\frac{2\pi^{d/2}}{\alpha\tau_*\Gamma(d/2)}}, \tag{5}$$

which coincides with Eq. (18) of the main text, up to the rate τ^{-1}, changed to τ_*^{-1}.

Maximal number of engrams for space correlated stimuli

Let $K(\mathbf{r}_1',\mathbf{r}_1)$ be the correlation function for the successive hits. That is, $K(\mathbf{r}_1',\mathbf{r}_1)d\mathbf{r}_1'd\mathbf{r}_1dt$ gives the probability that if the small area $d\mathbf{r}_1$ around the point $\mathbf{r}_1$ has been hit, the successive hit point during the time interval dt would be in the area $d\mathbf{r}_1'$ around the point $\mathbf{r}_1'$. Then

$$\int d\mathbf{r}_1 \int_{\mathbf{r}_1'\in A_{tot}} K(\mathbf{r}_1',\mathbf{r}_1)\frac{dt}{\tau(\mathbf{r}_1')A_*}d\mathbf{r}_1'$$

gives the probability that a hit will be delivered to some of the existing engram during the time interval dt. Here the integration is performed over all possible locations of the preceding hit at point $\mathbf{r}_1$ and over all possible points of the successive hit $\mathbf{r}_1'$, which belong to the area of existing engrams. Then the generalization of the mean-field Eq. (4) reads,

$$\frac{d}{dt}A_{tot} \simeq \alpha N e^{-A_{tot}/(NA_{\max})} - \int d\mathbf{r}_1 \int_{\mathbf{r}_1'\in A_{tot}} K(\mathbf{r}_1',\mathbf{r}_1)\frac{1}{\tau(\mathbf{r}_1')A_*}d\mathbf{r}_1'\left[m\frac{A_{tot}}{N}-A_0\right]. \tag{6}$$

Looking again for a steady state, $dA_{tot}/dt = 0$, we assume that $A_0 \ll A_{tot}(\mathbf{r})/N(\mathbf{r})$ and $A_{\max} \gg A_{tot}(\mathbf{r})/N(\mathbf{r})$ and take into account that $m=1$. This yields,

$$N^2 \simeq \frac{A_{tot}}{\alpha A_*}\int d\mathbf{r}_1 \int_{\mathbf{r}_1'\in A_{tot}(\mathbf{r})} \frac{K(\mathbf{r}_1',\mathbf{r}_1)}{\tau(\mathbf{r}_1')}d\mathbf{r}_1'$$

Again, taking into account the condition $A_{tot} \simeq A_*$ which holds true for $N = N_{max}$, we obtain,

$$N \simeq \sqrt{\frac{A_*}{\alpha\tau_*}}, \qquad \tau_*^{-1} \equiv \frac{1}{A_*}\int d\mathbf{r}_1 \int_{\mathbf{r}_1'\in A_*} K(\mathbf{r}_1',\mathbf{r}_1)\frac{1}{\tau(\mathbf{r}_1')}d\mathbf{r}_1',$$

which again leads to Eq. (5), or Eq. (18) of the main text with the effective rate τ_*^{-1}.

Maximal number of engrams for time and space correlated stimuli

Consider now the kinetic equation for the total engram area for the case of time-correlated stimuli. In the case of lacking correlations, the probability to receive a stimulus by engram of the area A (see Eq. 12 of the main text) reads $A(t)/\tau A_*$. For the case of time-correlated stimuli, we assume that action of a stimulus at time t, after the last hit at time t_0 has the following rate, $A(t_0)K(t-t_0)/\tau A_*$, where the memory function, $K(t)$ describes the ability of the system to respond to a stimulus. Here we quantify the probability to respond to a stimulus, rather than the appearance of the stimulus itself; τ_r characterizes the time needed for the system to recover from the previous stimulus. For instance, one can use the following memory function:

$$\frac{A(t_0)}{\tau A_*}K(t-t_0) = \frac{A(t_0)}{\tau A_*}\left(1-e^{-(t-t_0)/\tau_r}\right), \tag{7}$$

where τ_r is the relaxation time. For $t-t_0 \gg \tau_r$ the system completely recovers, while for $t-t_0 \ll \tau_r$, the response to a stimulus is blocked, since $K(t-t_0) \approx 0$. The according generalization of Eq. (15) then reads,

$$\frac{d}{dt}A_{tot}(t) \simeq \alpha N\, e^{-A_{tot}(t)/NA_{\max}} - \frac{1}{\tau A_*}A_{tot}(t_0)K(t-t_0)\left[m\left(\frac{A_{tot}(t)}{N}-A_0\right)\right], \tag{8}$$

with the same meaning of all the terms as in Eq. (15). The steady state in this systems implies the averaging over the time of the preceding stimulus t_0. Hence, assuming that $A_0 \ll A_{tot}/N$ and $A_{\max} \gg A_{tot}/N$ and using the averaging over t_0, we obtain for the steady state:

$$N^2 \approx \frac{A_{tot}^2}{\alpha\tau A_*}\int_{-\infty}^{t}K(t-t_0)P(t-t_0)dt_0, \tag{9}$$

where $P(t-t_0)$ gives the probability of the time difference between successive stimuli (here we assume that this function does not depend on the dimension), and we exploit the same reasoning as in derivation of Eq. (18). Performing the integration and using again the condition $A_{tot} = A_*$, we finally arrive at

$$N_{\max} \simeq \sqrt{\frac{2\pi^{d/2}}{\alpha\tau_*\Gamma(d/2)}}, \qquad \frac{1}{\tau_*} = \frac{1}{\tau}\int_{-\infty}^{t}K(t-t_0)P(t-t_0)dt_0 \tag{10}$$

which coincides with Eq. (18) of the main text, up to the renormalized rate $1/\tau$, which changes to $1/\tau_*$. For the simplest case of $P(t) = \tau_0^{-1}e^{-t/\tau_0}$, we obtain $\tau_*^{-1} = \tau^{-1}(1+\tau/\tau_0)^{-1}$

Estimate of the accuracy of Eq. (1) of the main text

To obtain some analytic estimates for the condition of the accuracy of Eq. (1) of the main text, we assume that at time $t=0$ an engram suffered a hit, so that its length becomes $l \approx l_0 \ll l_{max}$ – the average length after a hit. Then let this interval grow for the time t_1 without a hit with the rate approximated by $\alpha(1-l_0/l_{max}+\ldots) \simeq \alpha$, so at $t=t_1$ its length becomes

$$l(t_1) \simeq l_0 + \alpha t_1.$$

According to our model, the probability of zero hits till time $t=t_1$ reads,

$$P_0(t_1) \simeq \exp\left(-\frac{t_1 l(t_1)}{\tau l_*}\right) = \exp\left(-\frac{t_1(l_0+\alpha t_1)}{\tau l_*}\right).$$

then the probability that this segment would experience a hit at the time interval $(t_1, t+dt_1)$ may be written as

$$P_1(t_1)dt_1 \simeq P_0(t_1)\frac{dt_1 l(t_1)}{\tau l_*} = \exp\left(-\frac{t_1(l_0+\alpha t_1)}{\tau l_*}\right)\frac{dt_1(l_0+\alpha t_1)}{\tau l_*},$$

so that the probability that a hit segment has a length l' takes the form,

$$P(l') = \int_0^\infty P_1(t)\delta\left(l'-l(t)\right)dt_1 = \int_0^\infty \exp\left(-\frac{t_1(l_0+\alpha t_1)}{\tau l_*}\right)\frac{(l_0+\alpha t_1)}{\tau l_*}\delta\left(l'-(l_0+\alpha t_1)\right)dt_1 = \frac{l'}{\alpha\tau l_*}\exp\left\{-\frac{l'(l'-l_0)}{\alpha\tau l_*}\right\}.$$

Then one can easily estimates the probability that the length of an after-hit segment l is larger then the length of a pre-hit segment l' (recall that $l' > l_0$):

$$\begin{aligned} P(l > l') &\simeq \int_{l_0}^{l_{max}} P(l')dl' \int_{l'}^{l_{max}} l_0^{-1} e^{-l/l_0} dl \approx \int_{l_0}^{\infty} \frac{l'}{\alpha\tau l_*} \exp\left\{-\frac{l'(l'-l_0)}{\alpha\tau l_*}\right\} dl' \int_{l'}^{\infty} e^{-l/l_0} dl/l_0 \\ &= \int_{l_0}^{\infty} \frac{l'}{\alpha\tau l_*} \exp\left\{-\frac{l'(l'-l_0)}{\alpha\tau l_*} - \frac{l'}{l_0}\right\} dl' = \varepsilon^2 \int_1^{\infty} \xi e^{\varepsilon^2 \xi(\xi-1)} e^{-\xi} d\xi \approx \varepsilon^2 \int_1^{\infty} \xi e^{-\xi} = \frac{2}{e}\varepsilon^2 \sim \frac{l_0^2}{\alpha\tau l_*} \ll 1, \end{aligned}$$

where we introduce $\xi = l'/l_0$ and $\varepsilon^2 = l_0^2/(\alpha\tau l_*) \ll 1$.
